# Space-Based Focal Plane Ambiguous Measurement Ballistic Target MeMber Tracking

**DOI:** 10.3390/s18113996

**Published:** 2018-11-16

**Authors:** Wei Zhao, Shucai Huang, Wenhuan Cao

**Affiliations:** Air and Missile Defense College, Air Force Engineering University, Xi’an 710051, China; hsc67118@126.com (S.H.); WHCao666@163.com (W.C.)

**Keywords:** target tracking, space-based, focal plane, ambiguous measurement, member filter

## Abstract

Aimed at space-based passive detection and tracking of ballistic targets, a multi-target multi-Bernoulli (MeMber) filtering algorithm based on a focal plane ambiguous measurement model is proposed. The measurement error sources of space-based passive detection are analyzed. It is found that focal plane target tracking is the basis of space target tracking in the framework of the distributed data processing structure, and the main error of focal plane measurement is pixel resolution. Based on the above analysis, the focal plane ambiguous measurement model is established to replace the traditional measurement model and the generalized likelihood function is designed. Finally, the MeMber filter is modified based on ambiguous measurement and generalized likelihood function. The simulation experiment compares the tracking effect of a MeMber filter based on ambiguous measurement and traditional measurement, respectively. The filter based on ambiguous measurement achieves better results. It shows that ambiguous measurement is closer to reality and has more application value.

## 1. Introduction

The space-based infrared detection system is becoming the best way to solve the problem of ballistic target early warning with the development of thermal imaging and tracking technology [1,2,3,4]. It can track all the targets concerned and provide effective information support for defense systems, strategic counterattack, fire control and effect evaluation. Detection information is usually processed in a distributed structure. A single platform infrared sensor detects and tracks the target in its own field of view. The tracking results are output to the fusion node for stereo fusion tracking and then the target trajectory is obtained [5,6]. Therefore, two-dimensional focal plane tracking of the target image is the most important part and also the key to detection information processing.

Unlike ordinary video target tracking, space-based infrared detectors are aimed at ballistic targets. Ballistic targets are small targets in space-based high-orbit detection scenarios. In the background of high-orbit space-based detectors, the imaging size of long-range targets and their plume on the focal plane is no more than one pixel. This is the difficulty of target tracking in space-based infrared detection system.

References [6,7,8] have studied two-dimensional focal plane target tracking, and established the target motion model and the sensor measurement model; the probability hypothesis density filter was introduced to deal with the multi-target tracking problem in a clutter environment, and the multi-sensor data fusion problem was studied. In this paper, the measurement model of the sensor is reconstructed. Considering that the measurement is inaccurate and cannot be described using random noise, it should be modeled as an UGA (unambiguously generated ambiguous measurement) [9]. Different from the traditional measurement method, the ambiguous measurement is not a specific value containing random noise, but the approximate area where the target is located. In space-based focal plane measurement, the pixel where the target is located is obtained using ambiguous measurement, but it is not sure where the target is located in the pixel.

In space-based infrared detection scenarios, clutters on the focal plane composed of clouds, atmosphere, aerial vehicles, and other radiation sources will interfere with target tracking. To solve this problem, many filtering algorithms are proposed, such as the single hypothesis correlation (SHC), multiple hypothesis correlation (MHC), and compound hypothesis correlation (CHC), represented by joint probabilistic data correlation (JPDA) [10,11]. In the case of a large number of clutters, it is difficult to achieve target tracking with traditional filtering algorithms, but the multi-target Bayesian recursive filter constructed by Mahler can effectively solve the tracking problem in such scenarios. The implementation methods of multi-target Bayesian recursive filters include the probability hypothesis density filter (PHD) [12,13], the cardinalized probability hypothesis density filter (CPHD) [14,15], and the multi-target multi-Bernoulli filter (MeMBer) [16,17,18]. The execution methods include Gaussian mixture approximation (GM) [19,20,21] and Sequential Monte Carlo (SMC) [22,23,24,25,26,27,28,29]. In addition, Vo’s Cardinality Balanced MeMBer Filter (CBMeMBer) [30,31] corrects the potential estimation bias of the MeMBer filter. CBMeMBer does not require clustering and the computational complexity is less than CPHD, so this paper uses the CBMeMBer filter to track the space-based focal plane boost trajectory target.

In the first section, the measurement error of the space-based infrared detector is analyzed, the focal plane measurement is modeled, and the generalized likelihood function is given; in the second section, the reconstruction algorithm of the MeMBer filter is given based on the UGA measurement; and in the third section, the simulation results are compared to verify the effectiveness of the proposed method.

## 2. Detection Error Analysis and Focal Plane Measurement Modeling

Three main types of errors exist in space-based infrared detection: time error, LOS (line of sight) error, and satellite position error [32]. The amplitude of the time error is relatively small, and it can be gradually smaller with continuous measurement. The LOS error is mainly caused by the instability of satellite attitude and the measurement error of infrared radiation. In addition, the measurement error can also be caused by the detector noise, infrared refraction, and attenuation. The ephemeris of the satellite is not real-time, thus the position of the satellite is not accurate.

According to the distributed information processing system of the space-based optical detection system, the measurement object of the detector focal plane is the target image, the error source is the infrared radiation measurement error, and the position of the image on the two-dimensional focal plane is obtained; the fusion node inverts the position of the image on each two-dimensional focal plane into the position of the target in three-dimensional space. The error sources are the position, attitude, direction, and time errors of the sensors, and the trajectory of the target in three-dimensional space is obtained. Therefore, only the errors caused by infrared radiation measurement need to be considered in focal plane target tracking, which is mainly determined by the pixel resolution of the focal plane.

**Figure 1:**
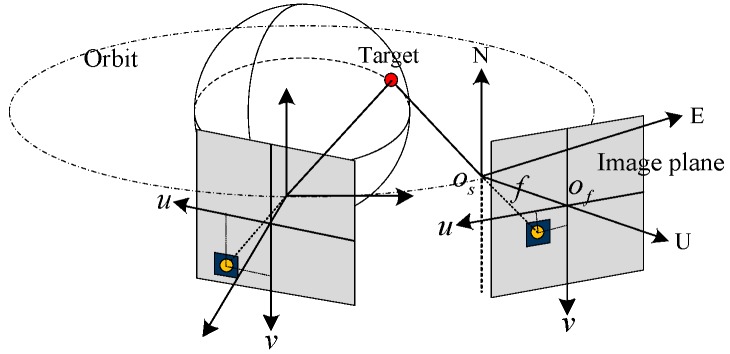
Coordinate system.

The relationship between the plane coordinate system and the sensor coordinate system is shown in Figure 1:

where $O_{f}-uv$ is the focal plane coordinate system, $O_{s}-UEN$ is the sensor coordinate system, and *f* is the focal length.

**Figure 2:**
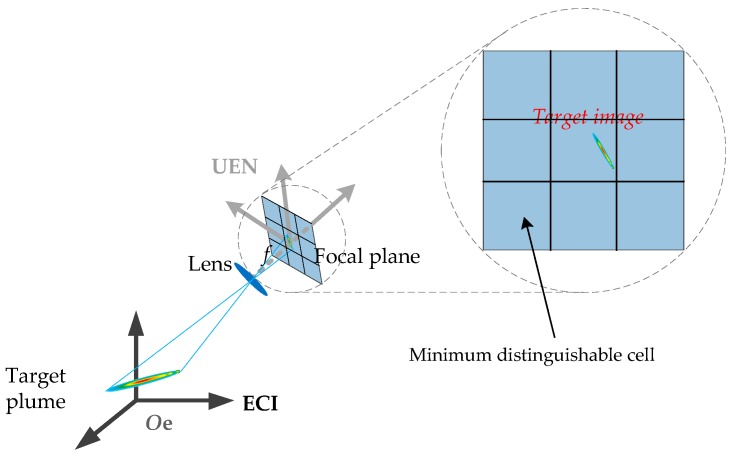
Minimum distinguishable cell.

For point targets, the error of infrared radiation measurement is mainly due to pixel resolution. As shown in Figure 2, in space-based infrared detection, when the target is too far away from the detector, its image occupies less than one pixel on the focal plane. By measuring the coordinates of the pixels, the position of the image cannot be given more accurately. The data we can obtain is which pixel the target is in. Therefore, it is unwise to use the center of the pixel as the target image position.

In traditional methods, observations are often modeled as noisy random variables.

(1) $$\left\{ \begin{array}{l} u=x+v_{x}\\ v=y+v_{y} \end{array}\right.$$

where $v_{x}$ and $v_{y}$ are zero mean Gaussian white noise $N(0,\sigma^{2})$ and the set of standard deviation σ needs to consider pixel resolution.

In fact, measurements should be modeled as follows:

The measurement is denoted by (u,v), the target position is denoted by (x,y), and the measurement equation is:

(2) $$\{\begin{array}{l} u=\left[\left\lfloor x/c\right\rfloor ,\left\lceil x/c\right\rceil \right]\\ v=\left[\left\lfloor y/c\right\rfloor ,\left\lceil y/c\right\rceil \right] \end{array}$$

In Equation (1), *u* and *v* are scalar; in Equation (2), *u* and *v* are interval variables represented by using upper and lower bounds, ‘⌊⌋’ is ‘Floor’, ‘⌈⌉’ is ‘Ceiling’, and *c* is pixel size.

## 3. MeMber Filter Based on Ambiguous Measurement

### 3.1. Generalized Likelihood Function Based on Ambiguous Measurement

The definition of generalized likelihood function given in Reference [33] is:

(3) f(Θ|x)≜Pr(η(x)∈Θ)

In the process of focal plane measurement, Θ is the area where the pixel is on the focal plane, and *x* denotes the location of the target.

**Figure 3:**
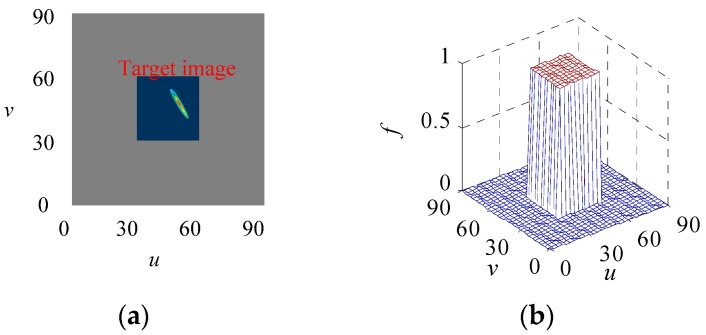
Likelihood probability density distribution. (**a**) is target image on focal plane, (**b**) is likelihood probability density distribution.

In other words, the generalized likelihood function is the probability of a target falling into a specified pixel region. As shown in Figure 3a, the target image is located in the dark area on the image plane. Due to the limitation of the pixel resolution, the accurate position of the target image cannot be obtained. Therefore, the likelihood probability density obeys a uniform distribution within the observation pixel region, and the probability density is 0 outside the observation area, as is shown in Figure 3b.

### 3.2. SMC-CBMeMber Filter Based on Ambiguous Measurement

**Figure 4:**
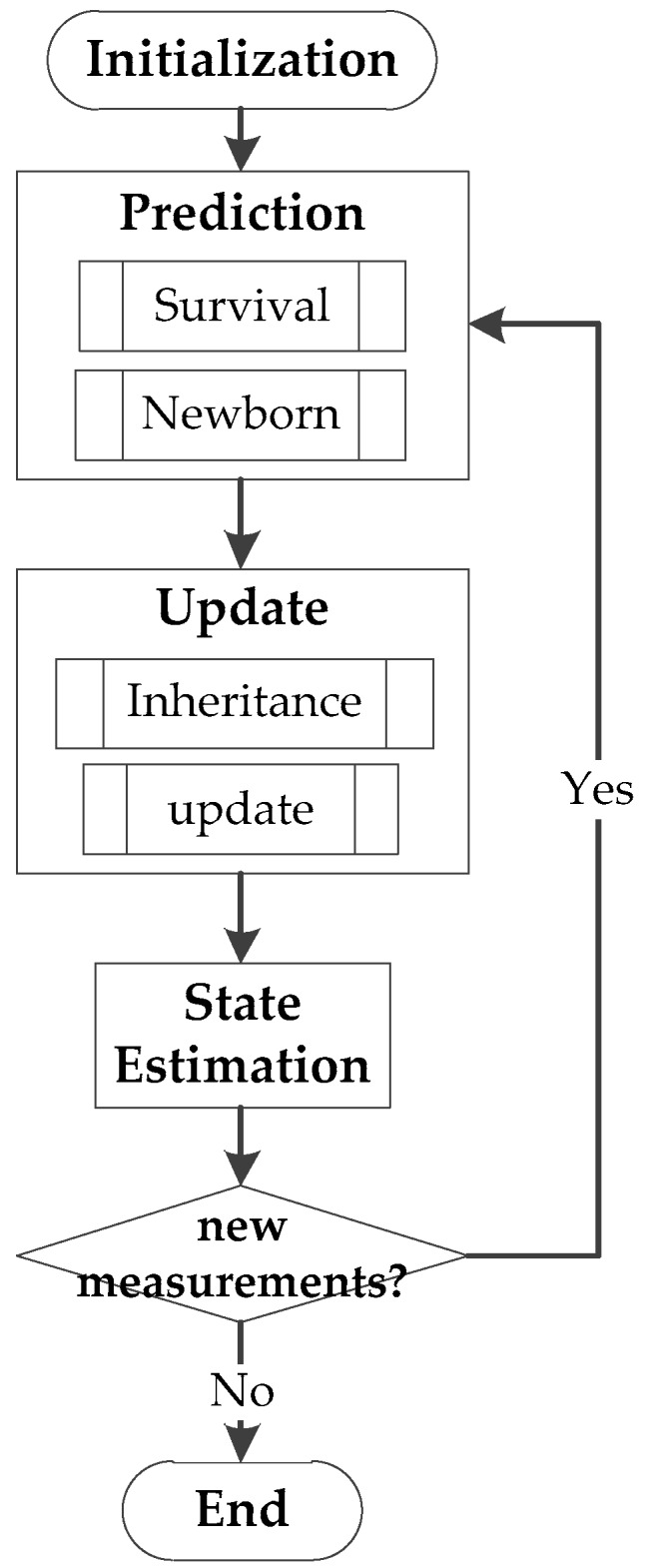
Block diagram of the CBMeMber filter.

The steps of the CBMeMber filter can be shown in a block diagram, as in Figure 4:

The two most crucial steps are the prediction and the update.

(1) Prediction

In time step *k* − 1, the posterior multi-target density is a multi-Bernoulli of the form:

(4) $$\pi_{k-1}={\left\{(r_{k-1}^{\left(i\right)},p_{k-1}^{\left(i\right)})\right\}}_{i=1}^{M_{k-1}}$$

The predicted multi-target density is also a multi-Bernoulli of the form:

(5) $$\pi_{k|k-1}={\left\{(r_{P,k|k-1}^{\left(i\right)},p_{P,k|k-1}^{\left(i\right)})\right\}}_{i=1}^{M_{k-1}}\cup {\left\{(r_{\Gamma ,k}^{\left(i\right)},p_{\Gamma ,k}^{\left(i\right)})\right\}}_{i=1}^{M_{\Gamma ,k}}$$

where ${\left\{(r_{\Gamma ,k}^{\left(i\right)},p_{\Gamma ,k}^{\left(i\right)})\right\}}_{i=1}^{M_{\Gamma ,k}}$ is the newborn MeMber density, ${\left\{(r_{P,k|k-1}^{\left(i\right)},p_{P,k|k-1}^{\left(i\right)})\right\}}_{i=1}^{M_{k-1}}$ is the survival MeMber density, and

(6) $$r_{P,k|k-1}^{\left(i\right)}=r_{k-1}^{\left(i\right)}\langle p_{k-1}^{\left(i\right)},p_{S,k}\rangle$$

(7) $$p_{P,k|k-1}^{\left(i\right)}(x)=\frac{\langle f_{k|k-1}(x|\cdot ),p_{k-1}^{\left(i\right)}p_{S,k}\rangle }{\langle p_{k-1}^{\left(i\right)},p_{S,k}\rangle }$$

(2) Update

The predicted multi-target density in time step *k* is denoted by

(8) $$\pi_{k|k-1}={\left\{(r_{k|k-1}^{\left(i\right)},p_{k|k-1}^{\left(i\right)})\right\}}_{i=1}^{M_{k|k-1}}$$

where $M_{k|k-1}=M_{k-1}+M_{\Gamma ,k}$;

In time step *k*, posterior multi-target density is a multi-Bernoulli of the form:

(9) $$\pi_{k}\approx {\left\{(r_{L,k}^{\left(i\right)},p_{L,k}^{\left(i\right)})\right\}}_{i=1}^{M_{k|k-1}}\cup {\left\{(r_{U,k}^{\left(i\right)}(z),p_{U,k}^{\left(i\right)}(x;z))\right\}}_{z\in Z_{k}}$$

where $Z_{k}$ is the measurement set in time step *k*, and ${\left\{(r_{L,k}^{\left(i\right)},p_{L,k}^{\left(i\right)})\right\}}_{i=1}^{M_{k|k-1}}$ is the inheritance (missing) track MeMber density. If the detection probability is set to $p_{D,k}(x)$, then

(10) $$r_{L,k}^{\left(i\right)}=r_{k|k-1}^{\left(i\right)}\frac{1-\langle p_{k|k-1}^{\left(i\right)},p_{D,k}\rangle }{1-r_{k|k-1}^{\left(i\right)}\langle p_{k|k-1}^{\left(i\right)},p_{D,k}\rangle }$$

(11) $$p_{L,k}^{\left(i\right)}=p_{k|k-1}^{\left(i\right)}(x)\frac{1-p_{D,k}(x)}{1-\langle p_{k|k-1}^{\left(i\right)},p_{D,k}\rangle }$$

${\left\{(r_{U,k}^{\left(i\right)}(z),p_{U,k}^{\left(i\right)}(x;z))\right\}}_{z\in Z_{k}}$ is the measurement updated MeMber density, and

(12) $$r_{U,k}^{\left(i\right)}(z)=\frac{\sum_{i=1}^{M_{k|k-1}}\frac{r_{k|k-1}^{\left(i\right)}(1-r_{k|k-1}^{\left(i\right)})\langle p_{k|k-1}^{\left(i\right)},\psi_{k,z}(x)\rangle }{{\left(1-r_{k|k-1}^{\left(i\right)}\langle p_{k|k-1}^{\left(i\right)},p_{D,k}\rangle \right)}^{2}}}{\kappa_{k}(z)+\sum_{i=1}^{M_{k|k-1}}\frac{r_{k|k-1}^{\left(i\right)}\langle p_{k|k-1}^{\left(i\right)},\psi_{k,z}(x)\rangle }{1-r_{k|k-1}^{\left(i\right)}\langle p_{k|k-1}^{\left(i\right)},p_{D,k}\rangle }}$$

(13) $$p_{U,k}^{\left(i\right)}(x;z)=\frac{\sum_{i=1}^{M_{k|k-1}}\frac{r_{k|k-1}^{\left(i\right)}}{1-r_{k|k-1}^{\left(i\right)}}p_{k|k-1}^{\left(i\right)}\psi_{k,z}(x)}{\sum_{i=1}^{M_{k|k-1}}\frac{r_{k|k-1}^{\left(i\right)}}{1-r_{k|k-1}^{\left(i\right)}}\langle p_{k|k-1}^{\left(i\right)},\psi_{k,z}\rangle }$$

(14) $$\psi_{k,z}(x)=g_{k}(z|x)p_{D,k}(x)$$

The SMC-CBMeMber filter is the particle realization form of CBMeMber, since the measurement involves only the MeMber density update section, the SMC-CBMeMber update section is briefly described.

The multi-target density at time step *k* − 1 is shown in Equation (4). Each component can be approximated by weighted particles $p_{k-1}^{\left(i\right)}(x)=\sum_{j=1}^{L_{k-1}^{\left(i\right)}}w_{k-1}^{\left(i,j\right)}\delta_{x_{k-1}^{\left(i,j\right)}}(x)$, where δ(x) is the Dirac function for the state ***x***. The prediction of each component is $p_{k|k-1}^{\left(i\right)}(x)=\sum_{j=1}^{L_{k|k-1}^{\left(i\right)}}w_{k|k-1}^{\left(i,j\right)}\delta_{x_{k|k-1}^{\left(i,j\right)}}(x)$.

The multi-target density of the inheritance (missing) part in the update step can be approximated to:

(15) $$w_{L,k}^{\left(i,j\right)}=\frac{w_{L,k-1}^{\left(i,j\right)}(1-p_{D,k}(x_{k|k-1}^{\left(i,j\right)}))}{\sum_{j=1}^{L_{k|k-1}^{\left(i\right)}}w_{k|k-1}^{\left(i,j\right)}(1-p_{D}(x_{k|k-1}^{\left(i,j\right)}))}$$

The measurement updated multi-target density ${\left\{(r_{U,k}^{\left(i\right)}(z),p_{U,k}^{\left(i\right)}(x;z))\right\}}_{z\in Z_{k}}$ can be approximated to:

(16) $$w_{U,k}^{\left(i,j\right)}(z)=\frac{w_{k|k-1}^{\left(i,j\right)}\frac{r_{k|k-1}^{\left(i\right)}}{1-r_{k|k-1}^{\left(i\right)}}\psi_{k,z}(x_{k|k-1}^{\left(i,j\right)})}{\sum_{i=1}^{M_{k|k-1}}\sum_{j=1}^{L_{k|k-1}^{\left(i\right)}}w_{k|k-1}^{\left(i,j\right)}\frac{r_{k|k-1}^{\left(i\right)}}{1-r_{k|k-1}^{\left(i\right)}}\psi_{k,z}(x_{k|k-1}^{\left(i,j\right)})}$$

The probability of existence $r_{L,k}^{\left(i\right)}$ and $r_{U,k}^{\left(i\right)}(z)$ can be calculated by substituting $p_{k|k-1}^{\left(i\right)}(x)=\sum_{j=1}^{L_{k|k-1}^{\left(i\right)}}w_{k|k-1}^{\left(i,j\right)}\delta_{x_{k|k-1}^{\left(i,j\right)}}(x)$ into Equations (9) and (11).

(17) $$\psi_{k,z}(x_{k|k-1}^{\left(i,j\right)})=\left\{ \begin{array}{ll} 1/c^{2}, & x_{k|k-1}^{\left(i,j\right)}\in z\\ 0, & x_{k|k-1}^{\left(i,j\right)}\notin z \end{array}\right.$$

## 4. Simulation Experiment

The following simulation experiments are designed to verify the effectiveness of the proposed ambiguous measurement MeMber filter in space-based infrared focal plane target tracking. To compare with the effect of the measurement modeling method based on Reference [2], the model of target motion is used.

(1) Motion modeling of boost-phase ballistic target

**Figure 5:**
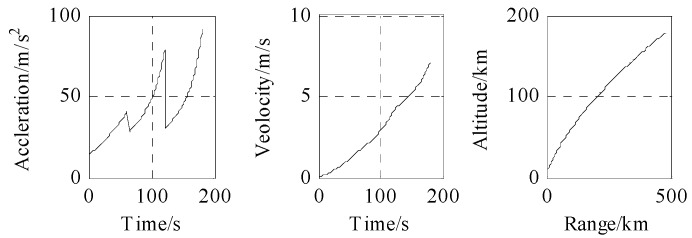
Motion modeling of boost phase.

**Figure 6:**
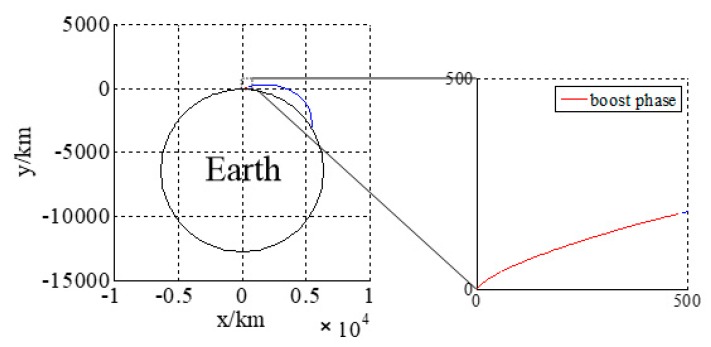
Trajectory in launch coordinate system.

The acceleration, velocity, and trajectory of the three-stage rocket model are shown in Figure 5. The trajectory in launch the coordinate system is shown in Figure 6: the red part is the boost phase, and the blue part is the free-flight phase. The total time is 180 s.

(2) Launch and detector parameter

Let us suppose that the satellite carrying the detector is a geosynchronous orbit satellite with a sub-satellite position (N 0°, W 40°), the sensor is a staring detector, the frame time is 1 second, pointing to the center of the earth, the focal plane array is 1024 × 1024 pixels, the pixel size is 30 μm × 30 μm, the focal length is 1 m, and the instantaneous field of view is 0.0307 rad × 0.0307 rad.

**Figure 7:**
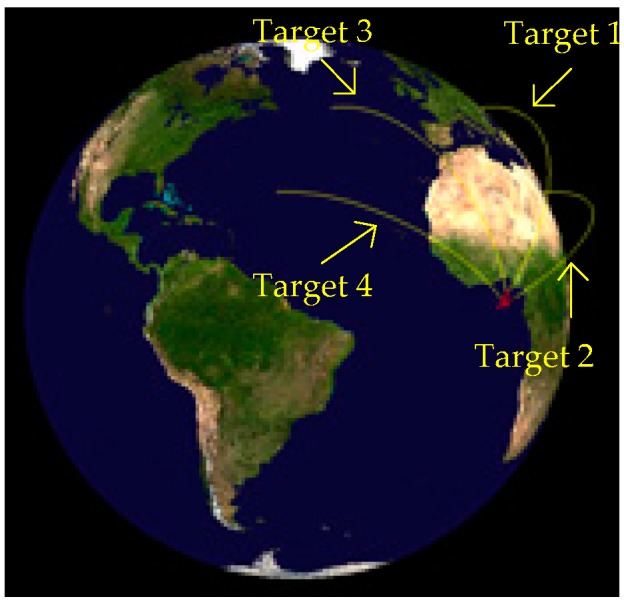
Trajectories (the red lines represent the boost phases of the ballistic missiles, and the yellow lines represent the free-flight phases).

**Figure 8:**
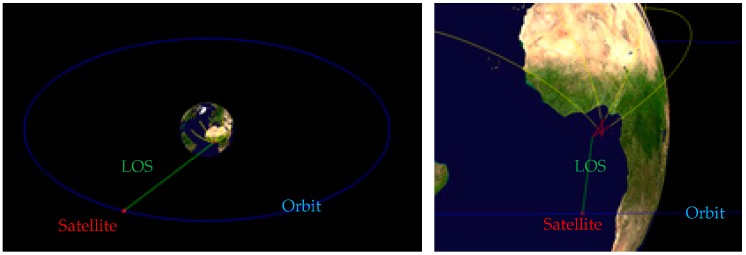
Detection scenario (the left picture is a panorama and the right one is a partial enlargement. The blue circle represents the detector orbit, the red ★ represents the detector, and the green line represents the line of sight).

**Figure 9:**
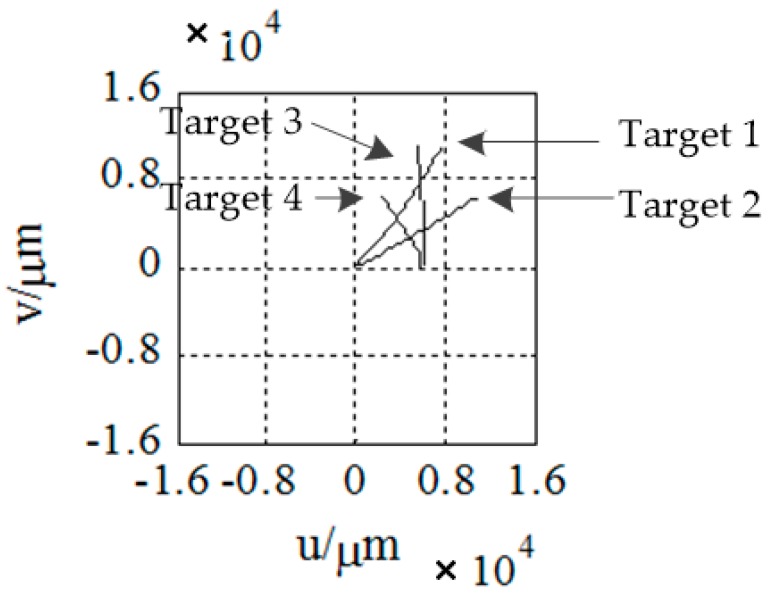
Traces of the targets on the focal plane.

As is shown in Figure 7 and Figure 8, four targets appear in the field of view, in turn, and the target motion conforms to the motion model established above. Let us suppose that all the targets are captured by the detector after 30 seconds of launching. Let us take the time when target 1 is captured as the initial time. The launch point and shooting are shown in Table 1. We then continue to monitor the field of view until all the targets burn out and the traces of the targets on the focal plane are obtained, as shown in Figure 9.

**Figure 10:**
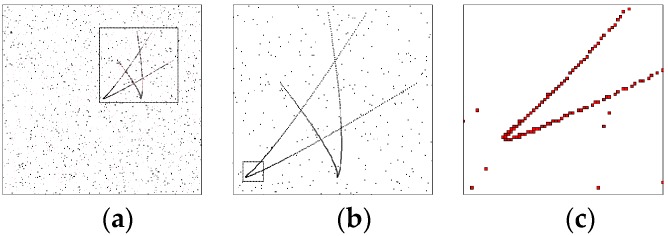
Measurements. (**a**) is all the measurements in the whole field of view, (**b**) is an enlargement of the dotted box in (**a**), (**c**) is an enlargement of the dotted box in (**b**).

We can assume that the number of clutters on the focal plane obeys a Poisson distribution with a parameter of 10, and all the 181 s measurements of the scene are shown in Figure 10. Figure 10a shows all the measurements in the whole field of view of the detector. To make the local details clearer, Figure 10b shows an enlargement of the dotted box in Figure 10a, and Figure 10c shows an enlargement of the dotted box in Figure 10b. The measurement is a rectangular region corresponding to the pixel, as can be seen clearly in Figure 10c.

(3) Filtering and tracking

The target tracking results obtained using the SMC-CBMeMber filter are as follows. The cardinality and optimal subpattern assignment (OSPA) are selected as evaluation indexes.

**Figure 11:**
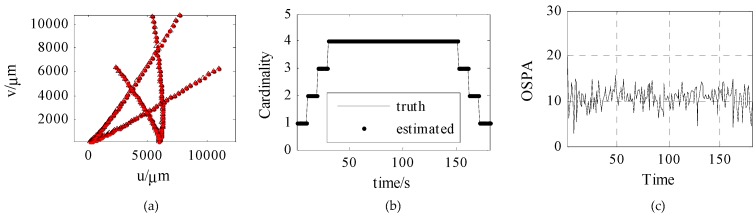
Trajectory tracking, cardinality estimated, and OSPA based on ambiguous measurement. (**a**) is trajectory tracking, (**b**) is cardinality estimated, (**c**) is OSPA.

Figure 11 shows the trajectory tracking curves, estimated cardinality, and OSPA based on ambiguous measurement. Among them, Figure 11a is the trajectory tracking effect, where the red dot represents the real target trajectory, and the triangle represents the estimated target trajectory; Figure 11b is the estimated cardinality of 181 s; Figure 11c is the OSPA of 181 s. As can be seen from the Figure, the accurate target trajectory and target number can be obtained by filtering, and the OSPAs are all smaller than 20.

**Figure 12:**
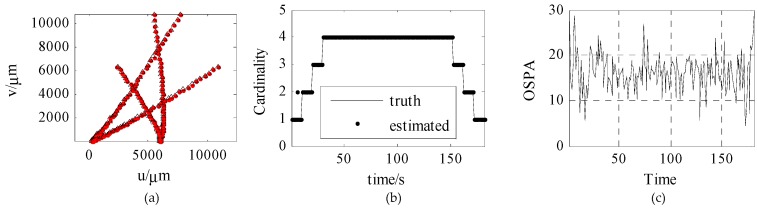
Trajectory tracking, cardinality estimated, and OSPA based on traditional measurement. (**a**) is trajectory tracking, (**b**) is cardinality estimated, (**c**) is OSPA.

Figure 12 shows the trajectory tracking curves, estimated cardinality, and OSPA based on traditional measurement. As can be seen from the Figure, the SMC-CBMeMber filter based on traditional measurement has a relatively large trajectory tracking error, a slight deviation in cardinality estimation, and a relatively large OSPA.

In general, the SMC-CBMeMber filtering method based on ambiguous measurement achieves better results, with a more accurate trajectory tracking effect and a more accurate target number estimation.

The reason for this is that the measurement noise is modeled as a Gaussian distribution in traditional measurement; as such, the likelihood probability is distributed across the whole observation space, meaning that invalid observation will have some side effects on the target trajectory estimation. The likelihood probability of ambiguous measurement, on the other hand, is only distributed in part of the space where the observation pixel is located, meaning that the estimation of the target trajectory is not affected by the observation of the far away particles set.

## 5. Conclusions

In this paper, a multi-target tracking method for the space-based infrared focal plane in clutter condition is studied, and an ambiguous measurement model is established to model the measurement as an interval variable, which is more in line with the actual situation. Finally, the SMC-CBMeMber filtering algorithm based on ambiguous measurement shows better results in simulation. In the future, further research will be carried out in relation to three aspects: first, the detection environment and clutter density will be studied in depth to improve the reliability of the algorithm; second, interval analysis theory will be introduced to improve the tracking algorithms; and third, ground experiments and environmental simulation experiments will be gradually carried out.

## Figures and Tables

**Table 1:** Launch parameters.

	Target 1	Target 2	Target 3	Target 4
Launch point	(N 0°, E 0°)	(N 0°, E 0°)	(N 0°, E 3°)	(N 0°, E 3°)
Shooting	30°	60°	330°	300°
Captured time	T0	T0 + 10 s	T0 + 20 s	T0 + 30 s
